# Supplementary material for: Behavioral flexibility and problem solving in an invasive bird
Source: PeerJ. 2016 May 3;4:e1975. doi: 10.7717/peerj.1975 (PMC4860340; doi:10.7717/peerj.1975)
Supplement: Table S1 — The order in which the more functional heavy (dark gray) or less functional light (light gray) objects were inserted (columns) into a water tube and whether the bird successfully obtained the food (marked with an X) for trials 1–20 (rows). Choices where objects were accidentally inserted into the tube are indicated by a black border. Accidents were kept in the analyses because they still provided an opportunity for the bird to learn something about the task. [file peerj-04-1975-s001.docx]

| **Tequila** | | | | | | **Margarita** | | | | | | | **Cerveza** | | | | | | | **Batido** | | | | | | **Horchata** | | | | **Refresco** | | | | | | | |
| --- | --- | --- | --- | --- | --- | --- | --- | --- | --- | --- | --- | --- | --- | --- | --- | --- | --- | --- | --- | --- | --- | --- | --- | --- | --- | --- | --- | --- | --- | --- | --- | --- | --- | --- | --- | --- | --- |
|  | **Insertion Order** | | | | |  | **Insertion Order** | | | | | |  | **Insertion Order** | | | | | |  | **Insertion Order** | | | | |  | **Insertion Order** | | |  | **Insertion Order** | | | | | | |
| **Trial** | 1 | 2 | 3 | 4 | 5 | **Trial** | 1 | 2 | 3 | 4 | 5 | 6 | **Trial** | 1 | 2 | 3 | 4 | 5 | 6 | **Trial** | 1 | 2 | 3 | 4 | 5 | **Trial** | 1 | 2 | 3 | **Trial** | 1 | 2 | 3 | 4 | 5 | 6 | 7 |
| 1 | X |  |  |  |  | 1 |  |  |  |  |  |  | 1 |  |  |  |  |  |  | 1 |  | X |  |  |  | 1 |  |  | X | 1 |  | X |  |  |  |  |  |
| 2 |  |  | X |  |  | 2 | X |  |  |  |  |  | 2 |  |  | X |  |  |  | 2 |  |  | X |  |  | 2 |  |  |  | 2 | X |  |  |  |  |  |  |
| 3 |  |  | X |  |  | 3 | X |  |  |  |  |  | 3 |  | X |  |  |  |  | 3 |  |  |  | X |  | 3 |  | X |  | 3 | X |  |  |  |  |  |  |
| 4 |  |  | X |  |  | 4 | X |  |  |  |  |  | 4 | X |  |  |  |  |  | 4 |  |  |  | X |  | 4 |  | X |  | 4 |  |  | X |  |  |  |  |
| 5 |  |  |  | X |  | 5 |  |  |  | X |  |  | 5 |  |  | X |  |  |  | 5 |  |  | X |  |  | 5 | X |  |  | 5 |  |  |  |  | X |  |  |
| 6 | X |  |  |  |  | 6 | X |  |  |  |  |  | 6 |  |  | X |  |  |  | 6 |  | X |  |  |  | 6 |  | X |  | 6 | X |  |  |  |  |  |  |
| 7 |  |  |  | X |  | 7 |  | X |  |  |  |  | 7 |  | X |  |  |  |  | 7 |  |  |  |  | X | 7 | X |  |  | 7 |  | X |  |  |  |  |  |
| 8 |  |  |  |  | X | 8 | X |  |  |  |  |  | 8 |  |  | X |  |  |  | 8 |  |  |  | X |  | 8 |  | X |  | 8 |  | X |  |  |  |  |  |
| 9 |  | X |  |  |  | 9 |  |  |  |  | X |  | 9 |  |  |  |  |  | X | 9 |  |  | X |  |  | 9 |  | X |  | 9 |  |  |  |  |  |  |  |
| 10 |  | X |  |  |  | 10 |  |  | X | X |  |  | 10 | X |  |  |  |  |  | 10 | X |  |  |  |  | 10 |  |  | X | 10 |  | X |  |  |  |  |  |
| 11 |  |  |  |  |  | 11 | X | X |  |  |  |  | 11 |  |  |  |  |  |  | 11 | X |  |  |  |  | 11 | X |  |  | 11 |  |  | X | X |  |  |  |
| 12 | X |  |  |  |  | 12 | X | X |  |  |  |  | 12 |  |  |  |  | X |  | 12 |  | X |  |  |  | 12 |  |  |  | 12 |  |  |  |  | X |  |  |
| 13 | X |  |  |  |  | 13 |  | X |  |  |  |  | 13 | X |  |  |  |  | X | 13 |  |  | X |  |  | 13 | X |  |  | 13 |  |  |  |  |  | X |  |
| 14 |  |  |  | X |  | 14 |  | X |  | X |  |  | 14 | X |  |  |  |  |  | 14 |  |  |  |  |  | 14 |  |  |  | 14 |  |  | X |  |  |  |  |
| 15 |  | X |  |  |  | 15 |  |  |  |  |  | X | 15 |  | X |  |  |  |  | 15 |  |  |  |  |  | 15 |  |  |  | 15 |  |  | X |  |  |  |  |
| 16 | X |  |  |  |  | 16 |  |  |  |  | X |  | 16 | X |  | X |  |  |  | 16 |  | X |  |  |  | 16 |  |  |  | 16 |  |  | X |  |  |  |  |
| 17 | X |  |  |  |  | 17 |  | X |  |  |  |  | 17 |  |  | X |  |  |  | 17 |  | X |  |  |  | 17 |  |  |  | 17 |  |  |  | X |  |  |  |
| 18 |  | X |  |  |  | 18 | X |  |  |  |  |  | 18 |  |  | X |  |  |  | 18 | X |  |  |  |  | 18 | X |  |  | 18 |  |  |  |  |  |  |  |
| 19 | X |  |  |  |  | 19 | X |  |  |  |  |  | 19 |  |  | X |  |  |  | 19 |  |  |  |  |  | 19 | X |  |  | 19 |  |  |  | X |  |  |  |
| 20 | X |  |  |  |  | 20 |  | X |  |  |  |  | 20 |  |  | X |  |  |  | 20 |  |  |  |  |  | 20 |  |  |  | 20 |  |  |  |  |  | X |  |

**Table 4. Heavy vs. Light:** the order in which the more functional heavy (dark gray) or less functional light (light gray) objects were inserted (columns) into a water tube and whether the bird successfully obtained the food (marked with an X) for trials 1-20 (rows). Choices where objects were accidentally inserted into the tube are indicated by a black border. Accidents were kept in the analyses because they still provided an opportunity for the bird to learn something about the task.
