# Supplementary material for: Behavioral flexibility and problem solving in an invasive bird
Source: PeerJ. 2016 May 3;4:e1975. doi: 10.7717/peerj.1975 (PMC4860340; doi:10.7717/peerj.1975)
Supplement: Table S3 — The order in which objects were inserted (columns) into functional narrow (dark gray) or non-functional wide (light gray) water tubes and whether the bird successfully obtained the food (marked with an X) for trials 1–20 (rows). Choices where objects were accidentally inserted into the tube are indicated by a black border. Accidents were kept in the analyses because they still provided an opportunity for the bird to learn something about the task. Note that Cerveza and Refresco obtained food from the wide tube twice because their motivation changed unexpectedly, thus changing their reachable distance. [file peerj-04-1975-s003.docx]

| **Cerveza** | | | | | **Batido** | | | | | **Refresco** | | | | |
| --- | --- | --- | --- | --- | --- | --- | --- | --- | --- | --- | --- | --- | --- | --- |
|  | **Insertion Order** | | | |  | **Insertion Order** | | | |  | **Insertion Order** | | | |
| **Trial** | 1 | 2 | 3 | 4 | **Trial** | 1 | 2 | 3 | 4 | **Trial** | 1 | 2 | 3 | 4 |
| 1 |  |  |  |  | 1 | X |  |  |  | 1 | X |  |  |  |
| 2 |  |  |  |  | 2 |  |  | X |  | 2 |  | X |  |  |
| 3 |  |  |  |  | 3 | X |  |  |  | 3 |  |  |  |  |
| 4 |  |  |  |  | 4 |  |  |  |  | 4 |  |  |  |  |
| 5 |  |  |  |  | 5 |  |  |  |  | 5 |  | X |  |  |
| 6 |  |  |  |  | 6 | X |  |  |  | 6 | X |  |  |  |
| 7 |  |  |  |  | 7 | X |  |  |  | 7 |  |  |  |  |
| 8 |  | X |  |  | 8 |  | X |  |  | 8 | X |  |  |  |
| 9 | X |  |  |  | 9 |  | X |  |  | 9 | X |  |  |  |
| 10 |  |  |  | X | 10 | X |  |  |  | 10 | X |  |  |  |
| 11 |  | X |  |  | 11 | X |  |  |  | 11 |  |  |  |  |
| 12 |  | X |  |  | 12 |  | X | X |  | 12 |  |  |  |  |
| 13 |  |  |  | X | 13 |  | X |  |  | 13 |  | X |  |  |
| 14 |  | X |  |  | 14 | X |  |  |  | 14 |  |  |  |  |
| 15 |  |  |  | X | 15 | X |  |  |  | 15 |  |  | X |  |
| 16 |  | X |  |  | 16 |  |  | X |  | 16 |  |  |  | X |
| 17 |  |  |  | X | 17 | X |  |  |  | 17 |  |  |  |  |
| 18 |  |  |  |  | 18 |  |  | X | X | 18 |  | X |  |  |
| 19 |  | X |  |  | 19 |  | X |  |  | 19 |  |  | X |  |
| 20 |  |  |  |  | 20 |  |  | X |  | 20 |  | X |  |  |

**Table 6. Narrow vs. Wide Equal Water Levels:** the order in which objects were inserted (columns) into functional narrow (dark gray) or non-functional wide (light gray) water tubes and whether the bird successfully obtained the food (marked with an X) for trials 1-20 (rows). Choices where objects were accidentally inserted into the tube are indicated by a black border. Accidents were kept in the analyses because they still provided an opportunity for the bird to learn something about the task. Note that Cerveza and Refresco obtained food from the wide tube twice because their motivation changed unexpectedly, thus changing their reachable distance.
